# *B7-H4 *gene polymorphisms are associated with sporadic breast cancer in a Chinese Han population

**DOI:** 10.1186/1471-2407-9-394

**Published:** 2009-11-11

**Authors:** Jie Zhang, Mingyan Zhang, Wei Jiang, Lihong Wang, Zhenkun Fu, Dalin Li, Da Pang, Dianjun Li

**Affiliations:** 1 Department of Immunology, Harbin Medical University, Harbin, Heilongjiang, PR China; 2 Department of Bioinformatics, Harbin Medical University, Harbin, Heilongjiang, PR China; 3 Institute of Cancer Prevention and Treatment, Harbin Medical University, Harbin, Heilongjiang, PR China; 4 Department of Surgery, the Third Affiliated Hospital of Harbin Medical University, Harbin, Heilongjiang, PR China

## Abstract

**Background:**

B7-H4, a co-inhibitory molecule of the B7 family, can restrain T cell proliferation, cytokine secretion and the development of cytotoxicity. B7-H4 is expressed in tumor tissues at a higher level than in normal tissues, and has a potential effect to protect tumors from anti-tumor immune responses. This case-control study was carried out to determine the potential influences of *B7-H4 *gene polymorphisms on the susceptibility and progression of breast cancer in Han women of Northeast China.

**Methods:**

We genotyped three *B7-H4 *variants (rs10754339, rs10801935 and rs3738414) and tagged all common haplotypes (frequency greater than or equal to 1%) in a Chinese population consisting of 500 breast cancer cases and 504 control individuals matched for age. Polymerase chain reaction-restriction fragment length polymorphism (PCR-RFLP) technique was used to determine the genotypes.

**Results:**

Our data indicated that, compared with the common genotype and allele of each SNP, the rs10754339 AG genotype and G allele showed a significantly increased risk of breast cancer (OR = 1.455, 95% CI 1.119-1.892; OR = 1.325, 95% CI 1.073-1.637, respectively). The rs10801935 CC genotype, the rs3738414 AA genotype and the rs3738414 A allele were associated with a significantly decreased risk of breast cancer (OR = 0.328, 95% CI 0.145-0.739; OR = 0.412, 95% CI 0.203-0.835; OR = 0.698, 95% CI 0.564-0.864, respectively). Additionally, the rs10754339 GG genotype was significantly associated with lymph node metastasis and PR status, and the G allele and the AG genotype were respectively associated with lymph node metastasis and ER status. In haplotype analysis, we observed that compared with the AAG haplotype, the AAA haplotype showed a significantly decreased risk of breast cancer (OR = 0.689, 95% CI 0.539-0.881), but the GAG haplotype was associated with a significantly increased risk of breast cancer (OR = 1.511, 95% CI 1.125-2.031). And the AAA and the GCG haplotypes also respectively have significant influences on tumor size and ER status.

**Conclusion:**

These results suggest that *B7-H4 *gene polymorphism may contribute to the sporadic breast cancer risk and prognosis in Chinese Han women.

## Background

Breast cancer is one of the most common malignant tumors in females, and its etiology as well as prognosis is extremely complex. The immune system, which plays an important role of immune surveillance in finding and eliminating cancer cells, can influence the development and growth of breast cancer. The central regulator of anti-tumor immune response is mediated by T lymphocyte, whose activation is regulated by co-stimulatory molecules, especially the receptors and ligands in B7-CD28 family [1,2].

B7-H4 is a recently identified member of B7 family, and conducts a co-inhibitory signal to T cell activation, which can restrain T cell proliferation, cytokine secretion and the development of cytotoxicity [3-5]. Experiments in vitro show that B7-H4 inhibits T cell activation through restraining IL-2 production and arresting cell cycles of both CD4^+ ^and CD8^+ ^T cell, and experiments in vivo also support the function of B7-H4 as an inhibitor to T cell mediated immunity [3-5]. *B7-H4 *mRNA transcriptions are detected extensively in spleen, lung, thymus and other normal tissues, but the proteins are not detectable in these tissues [3]. In contrast, high levels of B7-H4 protein are expressed in most kinds of tumors, such as breast cancer [6,7], ovarian cancer [7,8], lung cancer [8,9] and renal cell carcinoma [10]. Previous reports [6,7,11] showed that the high expression of B7-H4 protein in breast cancer decreased the number of tumor infiltrating lymphocytes (TILs) and prevented tumor cells from apoptosis. Therefore, the B7-H4 protein is a negative regulator of the anti-tumor immune response and may play an important role in breast cancer. And in our research, we investigated the association between *B7-H4 *gene polymorphisms and the risk of breast cancer.

The human *B7-H4 *gene is located on chromosome 1p11.1, and consists of six exons and five introns [8]. There are two types of *B7-H4 *mRNA transcriptions which are ~2.0 kb and ~800 bp. The larger transcription is dominant, and differs from the small one that has part of 3'-untranslating region (UTR) spliced. It is likely that the difference of 3'-UTR between the two transcripts affects the efficiency of B7-H4 protein synthesis [8]. In addition, *B7-H4 *mRNA transcription is inconsistent with its protein expression [3], indicating that B7-H4 expression is tightly controlled at the translational level in peripheral tissues. It has been pointed out that UTRs and introns, especially intron1, can modulate gene expression at many levels, such as the production of stable mRNA, the translational efficiency, the rate of mRNA decay, and so on [12-14]. In our study, we detected three polymorphic sites in UTRs and intron1, and established the association between *B7-H4 *gene polymorphisms and the risk of breast cancer in a Chinese Han population of Northeast China.

## Methods

### Subjects

The blood samples of the cases in our study were provided by the Department of Abdominal Surgery (The Third Affiliated Hospital of Harbin Medical University). They were collected from 500 Chinese women with sporadic breast cancer (mean age ± SD: 46.2 ± 7.6 years), and medical records were used to further confirm their pathological and clinical information of diagnoses (table 1). The blood samples of controls were contributed by 504 healthy women volunteers with matched ages who were from the same district (mean age ± SD: 43.0 ± 7.2 years). They were all selected randomly without any history of personal and familial malignancy or autoimmune diseases. All of the cases and healthy controls were recruited from Heilongjiang province of China during 2007 and 2008. Before the beginning of this study, ethical board approval from the Third Affiliated Hospital of Harbin Medical University was obtained, and each of the cases and controls signed the written informed consent.

**Table 1:** Clinicopathologic features of breast cancer cases (n = 500)

Feature		No. of cases (%)
Tumor type	IDC	426 (85.2)
	DCIS	29 (5.8)
	Other	45 (9.0)
Tumor size (cm)	TZ ≤ 2	181 (36.2)
	2<TZ ≤ 5	219 (43.8)
	TZ>5	26 (5.2)
	Missing	74 (14.8)
LN involvement	Positive	220 (44.0)
	Negative	280 (56.0)
ER	Positive	241 (48.2)
	Negative	180 (36.0)
	Missing	79 (15.8)
PR	Positive	320 (64.0)
	Negative	99 (19.8)
	Missing	81 (16.2)

Abbreviations: DCIS = ductal carcinoma in situ; ER = estrogen receptor; IDC = infiltrative ductal carcinoma; LN = lymph node; PR = progesterone receptor; TZ = tumor size.

### SNP selection and genotype analysis

Using Pupasview software [15], we found that rs10754339 in 3'-UTR, and rs3738414 in 5'-UTR are located on exonic splicing enhancers (ESEs), which may influence splicing of the primary transcript with production of stable mRNAs [16]. We also found that rs10801935 in intron1 may cause the alteration of the pre-mRNA secondary structure as predicted by RNAstructure 4.5 software [17]. Therefore, we detected these three potential functional single nuclear polymorphisms (SNPs) (rs10754339, rs10801935 and rs3738414) to investigate their relevance to breast cancer in a Chinese Han population of Northeast China.

Genomic DNA was extracted from 5 ml frozen whole blood with the Universal Genomic DNA Extraction Kit Ver. 3.0 (TaKaRa, Japan), following the manufacturer's protocol. Then, through a PCR-RFLP assay, we determined the dimorphism of rs10754339, rs10801935 and rs3738414. The desired fragment was amplified by PCR with a Biometra T-Gradient Thermoblock (Biometra, Göttingen, Germany) in a reaction mixture of 25 μl solution containing 0.3 μg of genomic DNA template, 1.25 U of Taq DNA polymerase with 10×PCR buffer (Mg^2+ ^plus) and dNTPs contained in the kit (TaKaRa, Japan), and 0.1 μl of each primer (Invitrogen, China). PCR products were digested with restriction enzymes (NEB, UK) according to the manufacturer's description, and were analyzed with 2.0% agarose gel electrophoresis. Primer sequences of each SNPs were rs10754339 (F: 5'-TCCTATGGGTCTGTCAATG-3'; R: 5'-GCTGCTAAACTCAAAGGC-3'), rs10801935 (F: 5'-TAGTGGCGGTACAATAGC-3'; R: 5'-AGTGCCTCTGTTTCTTCC-3'), and rs3738414 (F: 5'-AAAGACCTCACTGCTGTTCC-3'; R: 5'-CCACAGTCAGGAGGAAAGTC-3'). Annealing temperatures were rs10754339 (53.2°C), rs10801935 (55.6°C), and rs3738414 (55.6°C). The lengths of PCR products were rs10754339 (341 bp), rs10801935 (466 bp), and rs3738414 (419 bp). And restriction enzymes were rs10754339 (MscI), rs10801935 (SalI), and rs3738414 (BtsI) (please refer to additional file 1). In order to ensure quality control of genotyping results, we randomly selected 10% of the samples to direct sequencing, and the results were consistent with the PCR-RFLP results.

### Statistical analysis

Hardy-Weinberg equilibrium (HWE) was tested using the genotyping results of cases and controls respectively. The polymorphisms were excluded if they deviated from HWE (P < 0.05), as well as if the minor allele frequency was less than 5% or the missing data was more than 25%. The frequencies of allele and genotype were obtained by a simple counting. Haploview 3.2 was used to reset haplotypes and evaluate the frequencies in this case-control study. Chi-square test was adopted using SPSS 13.0 software in order to compare the different distributions within the allele, genotype and haplotype frequencies in cases and controls respectively, and the threshold for significance was P < 0.05. 10,000 permutations were run by Haploview program to measure the P-value of allele frequencies after the correction for multiple-testing bias. An odds ratio (OR) and its 95% confidence interval (CI) were calculated using SPSS 13.0 software to estimate the relative risk associated with rare alleles for breast cancer.

## Results

### Genotype and allele frequencies

The *B7-H4 *gene polymorphisms were analyzed for 500 breast cancer cases and 504 healthy controls. Table 2 and 3 summarize respectively the genotype and the allele frequencies of the *B7-H4 *gene polymorphisms in cases and controls. For any of the three SNPs genotyped, there was no deviation from Hardy-Weinberg equilibrium (P > 0.05), and no minor allele frequency less than 5% or missing data more than 25%. The statistical results showed that, the genotype and allele distributions in rs10754339, rs10801935 and rs3738414 had statistically significant differences (details are shown in table 2 and 3). Compared with the AA genotype, the rs10754339 AG genotype showed a significantly increased risk of breast cancer (OR = 1.455, 95% CI 1.119-1.892, P = 0.005). And the rs10754339 G allele was associated with a significantly increased risk of breast cancer compared with the A allele (OR = 1.325, 95% CI 1.073-1.637, P = 0.009). The rs10801935 CC genotype was associated with a significantly decreased risk of breast cancer compared with the AA genotype (OR = 0.328, 95% CI 0.145-0.739, P = 0.005). In rs3738414, the AA genotype showed a significantly decreased risk of breast cancer compared with the GG genotype (OR = 0.412, 95% CI 0.203-0.835, P = 0.011), and the A allele was associated with a significantly decreased risk of breast cancer compared with the G allele (OR = 0.698, 95% CI 0.564-0.864, P = 0.001). Furthermore, we corrected P-values for multiple testing, and significant association was observed among the rs10754339 G allele and rs3738414 A allele (P = 0.036 and 0.003, respectively).

**Table 2:** Genotype frequencies of *B7-H4 *polymorphisms in cases and controls

Genotype		No. (%)		OR (95% CI)	P	Global P value^a^
					
		Cases (n = 500)	Controls (n = 504)			
rs10754339	AA	277 (55.40)	324 (64.29)	Reference		0.016
	AG	199 (39.80)	160 (31.75)	1.455 (1.119-1.892)	0.005	
	GG	24 (4.80)	20 (3.97)	1.404 (0.759-2.595)	0.278	
rs10801935	AA	344 (68.80)	338 (67.06)	Reference		0.017
	AC	148 (29.60)	142 (28.17)	1.024 (0.778-1.348)	0.865	
	CC	8 (1.60)	24 (4.76)	0.328 (0.145-0.739)	0.005	
rs3738414	GG	324 (64.80)	278 (55.16)	Reference		0.003
	AG	164 (32.80)	201 (39.88)	0.700 (0.539-0.909)	0.007	
	AA	12 (2.40)	25 (4.96)	0.412 (0.203-0.835)	0.011	

^a^Global P-value was calculated using Chi-square test.

Abbreviations: CI = confidence interval; OR = odds ratio.

**Table 3:** Allele counts and frequencies of *B7-H4 *polymorphisms in cases and controls

Allele		No. (%)		OR (95% CI)	P-value
				
		Cases (n = 500)	Controls (n = 504)		
rs10754339	A	753 (75.30)	808 (80.16)		
	G	247 (24.70)	200 (19.84)	1.325 (1.073-1.637)	0.009^a^
rs10801935	A	836 (83.60)	818 (81.15)	Reference	
	C	164 (16.40)	190 (18.85)	0.845 (0.671-1.063)	0.150
rs3738414	G	812 (81.20)	757 (75.10)	Reference	
	A	188 (18.80)	251 (24.90)	0.698 (0.564-0.864)	0.001^b^

^a^P = 0.036 and ^b^P = 0.003 after correcting P-value for multiple testing with 10,000 permutations by Haploview program.

Abbreviations: CI = confidence interval; OR = odds ratio.

In addition, we analyzed the correlation between the polymorphisms of *B7-H4 *gene and a series of clinicopathologic features, including lymph node metastasis, tumor size, and the statuses of estrogen receptor (ER) and progesterone receptor (PR). It was observed that in rs10754339, compared with the AA genotypes, the GG genotype was associated with a significantly increased risk of lymph node metastasis (OR = 2.492, 95% CI 1.054-5.894, P = 0.033), and the G allele showed a significantly increased risk of lymph node metastasis compared with the A allele (OR = 1.394, 95% CI 1.045-1.860, P = 0.024). Compared with the rs10754339 AA genotype, the AG genotype was associated with a significantly increased expression of ER (OR = 1.640 95% CI 1.092-2.464, P = 0.017), and the GG genotype was associated with a decreased expression of PR (OR = 0.367 95% CI 0.135-0.994, P = 0.041). However, no obvious correlation was revealed between the genotype and allele frequencies and tumor size.

### Haplotype analysis

The association between *B7-H4 *SNPs and breast cancer was further confirmed in our cohort by Haploview program. Possible haplotypes were then reconstructed, of which the ones with frequencies ≥ 1% are shown in table 4. The most frequently appeared haplotype in cases and controls were AAG (rs10754339A, rs10801935A, rs3738414G) (50.7%). And we found that compared with the AAG haplotype, the AAA haplotype showed a significantly decreased risk of breast cancer (OR = 0.689, 95% CI 0.539-0.881, P = 0.003), but the GAG haplotype was associated with a significantly increased risk of breast cancer (OR = 1.511, 95% CI 1.125-2.031, P = 0.006).

**Table 4:** *B7-H4 *haplotype frequencies in cases and controls

*B7-H4 *haplotype			**Freq**.	cases (n = 500)	controls (n = 504)	OR (95% CI)	P-value
					
rs10754339	rs10801935	rs3738414					
A	A	G	0.507	52.0%	49.5%	Reference	
A	A	A	0.173	14.5%	20.0%	0.689 (0.539-0.881)	0.003
G	A	G	0.112	13.7%	8.6%	1.511 (1.125-2.031)	0.006
A	C	G	0.090	8.3%	9.6%	0.821 (0.598-1.128)	0.224
G	C	G	0.073	7.2%	7.4%	0.934 (0.660-1.321)	0.698
G	A	A	0.032	3.4%	3.1%	1.052 (0.637-1.739)	0.842

Abbreviations: CI = confidence interval; OR = odds ratio.

We also analyzed the association between the haplotypes and the clinical features of cases. We found that the AAA haplotype was significantly associated with tumor size (P = 0.020), and compared with the AAG haplotype, the GCG haplotype frequency was associated with a significantly increased expression of ER (OR = 1.903, 95% CI 1.034-3.502, P = 0.036). However, no significant association was obtained between haplotypes and either the lymph node metastasis or the statuses of PR.

## Discussion

Breast cancer is one of the polygenetic disorders with complex inheritance patterns. Recently, the determination of genetic polymorphisms provided a new approach to investigate the etiology of such complex genetic diseases [18]. Reports indicate that breast cancer susceptibility and prediction can be influenced by the polymorphism of *cytotoxic T-lymphocyte antigen-4 *(*CTLA-4*) gene, which encodes a classic molecule offering a co-inhibitory signal to T cell activation [19]. Additionally, some other genes encoding novel co-inhibitors, such as *PD-1 *and *B and T lymphocyte attenuator (BTLA)*, are attractive in case-control studies, as candidate genes between the gene polymorphisms and the risk of diseases [20,21]. As another co-inhibitory molecule investigated more recently, B7-H4 has shown a potential effect on tumors, by which tumors may escape from an anti-tumor immune response. To elaborate, B7-H4 protein is over expressed in several malignant tumors while is little expressed in normal tissues [6-8,10], and its high expression in breast cancer decreased the number of TILs and prevented tumor cells from undergoing apoptosis [6,7,11].

In our case-control study, we chose three potentially functional polymorphisms of *B7-H4*, rs10754339, rs10801935 and re3738414, and firstly detected their association with the risk and prognosis of breast cancer in Chinese Han women. The results indicate that some of the alleles, genotypes and haplotypes of these three SNPs are associated with the risk and prognosis of breast cancer, which will be discussed below respectively. According to our results, in rs10754339, women with the AG genotype and G allele are likely to have an increased risk of breast cancer (OR = 1.455, 95% CI 1.119-1.892; OR = 1.325, 95%CI 1.073-1.637, respectively), indicating that the rs10754339 G allele maybe plays a risk role in breast cancer. It has been suggested that *B7-H4 *mRNA is widely distributed in lymphoid and nonlymphoid tissues. However, the protein is rarely expressed in normal tissues but highly expressed in many kinds of tumor tissues including breast cancer [3,6-10]. Also, 3'-UTR can regulate gene expression at many different levels, and cis-acting elements of 3'-UTR mediate the stability, degradation, and subcellular localization of mRNA [12,22]. Therefore, rs10754339 located on ESE may regulate the production of mRNA molecules through above mechanism, and G allele may be associated with an increased expression of B7-H4 and then influence the susceptibility of breast cancer. In addition, most tissues contain two types of *B7-H4 *transcriptions which are ~2.0 kb and ~800 bp. The larger one is major and their difference is that the small one has 3'-UTR spliced out partly [8]. Rs10754339 may also be involved in the formation and expression of different types of *B7-H4 *transcriptions.

In rs10801935, women with the CC genotype are likely to have a decreased risk of breast cancer (OR = 0.328, 95% CI 0.145-0.739), indicating that the rs10801935 CC genotype in intron 1 may be a protective factor in breast cancer. As we know, human gene expression requires transcription controls mediated by the presence of intron sequences, especially, the first intron, which contains various regulatory elements and splicing control elements [14,23]. Mutations occurring in introns can induce the aberrant splicing due to the disruption of the splice site, the splicing enhancers and silencers, or the alteration of the pre-mRNA secondary structure, which results in translational prevention [16,24,25]. Supposed to change the secondary structure of pre-mRNA, rs10801935 can possibly influence B7-H4 translational efficiency by causing deviant splicing, and CC genotype may down-regulate the B7-H4 expression and decrease the breast cancer risk.

Our research also suggests that women with the AA genotype and the A allele in rs3738414 are less likely to have breast cancer (OR = 0.412, 95% CI 0.203-0.835; OR = 0.698, 95% CI 0.564-0.864, respectively), indicating that the A allele of rs3738414 may have a potentially protective effect on breast cancer. 5'-UTR, upon which a number of DNA binding sites of regulatory factors exist, is important for post-transcriptional modification and translation efficiency [12]. Using Pupasview software [15], the rs3738414 is supposed to be located on ESE, and the mutation of rs3738414 perhaps changes the protein binding pattern, and further affects the B7-H4 expression. Consequently, rs3738414 A allele may reduce the B7-H4 expression and decrease the susceptibility of breast cancer.

In addition, according to the analysis for the association between *B7-H4 *gene polymorphisms and clinical presentations, we found that the rs10754339 GG genotype was significantly associated with lymph node metastasis and PR status, and the G allele and the AG genotype were respectively associated with lymph node metastasis and ER status. However, the other two SNPs in our study were found to be irrelevant to clinicopathologic features. Lymph node involvement, ER and PR status are important in predicting the long-term survival of breast cancer cases. Lymph node metastasis positive cases have higher mortality [26], and steroid hormone receptors are valuable as predictive markers of endocrine therapy [27]. Therefore, rs10754339 may be significant to forecast the prognosis of breast cancer.

The association between *B7-H4 *gene and breast cancer was further confirmed by the analysis of haplotype. Results suggested that the AAA haplotype showed a significantly decreased risk of breast cancer, whereas GAG haplotype had a contrary outcome (OR = 0.689, 95% CI 0.539-0.881; OR = 1.511, 95% CI 1.125-2.031). To elaborate, the AAA haplotype appears to be a protective factor in breast cancer, but the GAG haplotype may be a risk factor in breast cancer. Furthermore, we found that both AAA and GCG haplotypes had significantly correlations with the clinical presentations. These haplotypes may also be the valuable prognostic factors for survival of breast cancer cases.

## Conclusion

It was observed in our study that polymorphisms of the *B7-H4 *gene may affect the individual breast cancer risk and prognosis in Chinese Han women, which provides the first evidence for the involvement of the human *B7-H4 *gene in breast cancer. The variants of rs10754339, rs10801935 and rs3738414 are probably involved in the risk of breast cancer. Furthermore, rs10754339 may participate in the prognosis of breast cancer. AAA and GAG haplotypes probably influence on the breast cancer risk, and AAA and GCG may participate in the progression of breast cancer. However, studies focusing on new polymorphisms with more samples from other populations as well as the investigation of the basic functions of *B7-H4 *gene mutations remain to be further conducted.

## Competing interests

The authors declare that they have no competing interests.

## Authors' contributions

Jie Zhang performed the primer design and wrote the drafts. Jie Zhang, Mingyan Zhang, Lihong Wang, Zhenkun Fu and Dalin Li collected the case and control blood samples, performed the PCR-RFLP experiments. Wei Jiang contributed statistical analysis. Da Pang and Dianjun Li conceived of the study, participated in its design and coordination, and helped to draft the manuscript. All authors read and approved the final manuscript.

## Pre-publication history

The pre-publication history for this paper can be accessed here:

http://www.biomedcentral.com/1471-2407/9/394/prepub

## Supplementary Material

Additional file 1 **Supplemental Table - Primers and restriction enzymes of *B7-H4 *PCR-RFLP genotyping**. The data provided represent the primers and restriction enzymes used during *B7-H4 *PCR-RFLP genotyping process. Click here for file
