# Supplementary material for: B7-H4 gene polymorphisms are associated with sporadic breast cancer in a Chinese Han population
Source: BMC Cancer. 2009 Nov 11;9:394. doi: 10.1186/1471-2407-9-394 (PMC2780456; doi:10.1186/1471-2407-9-394)
Supplement: Additional file 1 — Supplemental Table - Primers and restriction enzymes of B7-H4 PCR-RFLP genotyping. The data provided represent the primers and restriction enzymes used during B7-H4 PCR-RFLP genotyping process. [file 1471-2407-9-394-S1.doc]

# Additional file 1

## Supplemental Table - Primers and restriction enzymes of *B7-H4* PCR-RFLP genotyping

| SNP ID | SNP primer sequence | T (°C) | Length (bp) | Restriction enzymes |
| --- | --- | --- | --- | --- |
| rs10754339 | F: 5′- TCCTATGGGTCTGTCAATG-3′ | 53.2 | 341 | MscI |
|  | R: 5′- GCTGCTAAACTCAAAGGC-3′ |  |  |  |
| rs10801935 | F: 5′-TAGTGGCGGTACAATAGC-3′ | 55.6 | 466 | SalI |
|  | R: 5′-AGTGCCTCTGTTTCTTCC-3′ |  |  |  |
| rs3738414 | F: 5′-AAAGACCTCACTGCTGTTCC-3′ | 55.6 | 419 | BtsI |
|  | R: 5′-CCACAGTCAGGAGGAAAGTC-3′ |  |  |  |

T = annealing temperature
